# Pyroptosis in the Initiation and Progression of Atherosclerosis

**DOI:** 10.3389/fphar.2021.652963

**Published:** 2021-05-26

**Authors:** Zhengtao Qian, Yilin Zhao, Chuandan Wan, Yimai Deng, Yaoyao Zhuang, Yeqiong Xu, Yanping Zhu, Shourong Lu, Zhengyang Bao

**Affiliations:** ^1^ Department of Clinical Laboratory, Changshu Medicine Examination Institute, Changshu, China; ^2^ Department of Internal Medicine, Affiliated Wuxi Matemity and Child Health Care Hospital of Nanjing Medical University, Wuxi, China; ^3^ Geriatric Department, Nanjing Medical University Afliated Wuxi People’s Hospital, Wuxi, China

**Keywords:** atherosclerosis, pyroptosis, endothelial cells, macrophages, vascular smooth muscle cells, inflammation

## Abstract

Pyroptosis, a newly discovered form of programmed cell death, is characterized by cell swelling, the protrusion of large bubbles from the plasma membrane and cell lysis. This death pathway is mediated by the pore formation of gasdermin D (GSDMD), which is activated by human caspase-1/caspase-4/caspase-5 (or mouse caspase-1/caspase11), and followed with the releasing of both cell contents and proinflammatory cytokines. Pyroptosis was initially found to function as an innate immune effector mechanism to facilitate host defense against pathogenic microorganisms, and subsequent studies revealed that pyroptosis also plays an eventful role in inflammatory immune diseases and tumor resistance. Recent studies have also shown that pyroptosis is involved in the initiation, the progression and complications of atherosclerosis. Here, we provide an overview of the role of pyroptosis in atherosclerosis by focusing on three important participating cells: ECs, macrophages, and SMCs. In addition, we also summarized drugs and stimuli that regulate the progression of atherosclerosis by influencing cell pyroptosis.

## Introduction

In recent decades, atherosclerotic cardiovascular disease was found to the main cause of vascular diseases, which is developed by the accumulation fatty and/or fibrous material in vascular intima. Endothelial cells (ECs), smooth muscle cells (SMCs) and macrophages are main cell types involved in the atherosclerosis. ECs and SMCs constitute the structure of vascular. ECs dysfunction and death induce the release of inflammatory cytokines and the monocytes recruitment, which is the initiation of atherosclerosis (Libby et al., 2019). It is worth noting that the infiltration of inflammatory cells in the arterial wall is a well-known important process of atherosclerosis (Chen et al., 2010). Inflammatory cells, consisted mainly of monocytes, are recruited by proinflammatory cytokines and chemokines that is released by damaged ECs and migrate to the injury site. As inflammation in subendothelial worsen, the incompletely differentiated SMCs proliferate and migrate to the endothelial, accelerate the progression of atherosclerosis (Choy et al., 2001; Savoia and Schiffrin, 2007). Recent experimental data suggest that macrophages and SMCs uptake low-Density Lipoprotein Cholesterol (LDL-C) in atherosclerotic plaque and lead to the plaque formation (Bennett et al., 2016a; Basatemur et al., 2019). Under physiological conditions, inflow and outflow of monocyte/macrophage in vascular has a balance, eliminate the inflammatory cytokines and metabolic waste in vascular (Curtiss, 2009). In the progression of atherosclerosis, macrophages and SMCs phagocytose excess lipid and their programmed death leads to the formation of necrotic cores (Geng and Libby, 1995; Clarke et al., 2010). These studies indicate that cell death and inflammation are two key factors in the initiation and progression of atherosclerosis.

As a membrane-pore-formation death mechanism, pyroptosis is closely related to the inflammatory response. Different from apoptosis and necrosis, pyroptosis has a unique mechanism. Pyroptosis is activated by pattern recognition receptors (PRR) and triggers inflammatory responses, which is part of innate immunity and is general in vertebrates (Jorgensen and Miao, 2015). After sensing ligands, the inflammasome assembles and cleaves caspase-1 (LPS sensing leads to activation of human caspase-4/5 or mouse caspase-11). The activated Caspase-1 or Caspase-4/5/11 cleaves GSDMD which forms cell membrane pores and eventually lead to pyroptosis. Caspase-1 also activate interleukin (IL)-1β and IL-18, and these cytokines along with other alarmins are secreted through cell membrane pores or after membrane lysis, which may cause the aggregation of immune cells (Shi et al., 2015). Pyroptsis changes the intracellular structure to kill intracellular bacteria and destroy the replication environment to resist pathogens, which exhibits a role of immune defense (Miao et al., 2010; Jorgensen et al., 2016). Moreover, excessive or poorly regulated cell death is increasingly recognized to contribute to chronic inflammatory disease. Pyroptosis can be both a consequence and a cause of inflammation, and these roles are difficult to be distinguished in chronic diseases (Anderton et al., 2020). Even so, increasing evidence has shown that pyroptosis may be involved in the pathological process of atherosclerosis (Chang et al., 2013; Xu et al., 2018). In this review, we summarize the function and influencing factors of pyroptosis in ECs, macrophages, and SMCs.

## Pyroptosis

In original cognition, pyroptosis was considered as a unique death form of monocytes, characterized by caspase-1 mediated and corresponding to certain bacterial stimulation. However, subsequent studies found that caspase-1 can also be activated in response to stimuli, such as infection and inflammatory factors, and induce pyroptosis in a variety of cells. With the discovery that caspase-11/4/5 induces pyroptosis by recognizing intracellular lipopolysaccharide (LPS), it was revealed that pyroptosis is not only specifically triggered by caspase-1. Based on this, pyroptosis-related cell death pathways are classified into two types: the caspase-1-mediated canonical inflammasome pathway and the human’s caspase-4/5 (or murine caspase-11)-mediated noncanonical inflammasome pathway. Recent studies have identified GSDMD, a substrate of caspase-1 and caspase-11/4/5, as the pyroptosis executioner. The gasdermin family proteins, including GSDMD exerts membrane pore-forming activity. Hence, pyroptosis has also been described as gasdermin-mediated programmed necrosis (Shi et al., 2017).

### Caspase-1 is Activated in the Canonical Inflammasome Pathway

In the canonical pathway of pyroptosis, the activation of caspase-1 triggered by activated inflammasomes leads to pyroptosis. Inflammasomes, which detect pathogen-associated molecular patterns (PAMPs) and danger-associated molecular patterns (DAMPs), consist of members of the nucleotide-binding oligomerization domain -like receptor (NLR) family and pyrin proteins (Rathinam et al., 2010; Heilig and Broz, 2018). Once PRR recognizes the signal of infection or immunological challenge, it will mediate the assembly of inflammasomes through downstream molecules. The assembled inflammasome complex binds to the precursor of caspase-1 (pro-caspase-1) and releases activated caspase-1, which eventually leads to pyroptosis (Broz and Dixit, 2016; Shi et al., 2017).

In the past decade, extensive studies have showed that specific types of inflammasomes recognize specific microbial stimuli and endogenous dangers (Broz and Dixit, 2016). The NOD-like receptor 3/apoptosis-associated speck-like proteins (NLRP3/ASC) inflammasome activating caspase-1 was first discovered by Kanneganti et al. (Kanneganti et al., 2006; Mariathasan et al., 2006; Sutterwala et al., 2006). As the most representative pyroptosis-related inflammasome in response to a variety of membrane damage signals, NLRP3 is involved in the pathological process of numerous inflammation-related diseases such as cancer, atherosclerosis, diabetes, and obesity (Sharma and Kanneganti, 2021). The absent in melanoma 2 (AIM2)/ASC inflammasome that recognize double-stranded DNA plays a role in pathogen infection. The NLR family of apoptosis inhibitory proteins (NAIP)/NLRC4 inflammasome can respond to Type III secretions and bacterial flagella. The NLRP1 inflammasome can specifically recognize Toxoplasma gondii infection and anthrax lethal toxin. The Pyrin/ASC inflammasome receive the signals of Rho GTPases inactivating modifications caused by bacterial toxins (Martinon et al., 2002; Zhao and Shao, 2016). After receiving the pyroptosis signal, NLRs, AIM2 and Pyrin bind to ASC with signaling domains. The bound ASC then recruits and cleaves the pro-caspase-1 to releases the activated caspase-1 (Aachoui et al., 2013a). Activated caspase-1 cleaves the linker of GSDMD and matures IL-1β/18, which is released to the outside of the cell through the membrane pore (Shi et al., 2015; Ding et al., 2016; Liu et al., 2016). Relevant studies on the inflammasome has showed that caspase-1 and caspase-1-mediated pyroptosis represent a very important defense mechanism in innate immune system (Shi et al., 2017).

### Caspase-11/4/5 is Activated in the Non-canonical Inflammasome Pathway

In the end of 2000s, studies have found that the death of mouse macrophages in response to Gram-negative bacteria infection is closely related to caspase-11 (Ng and Monack, 2013; Yang et al., 2015). Different from the Caspase-1-dependent death, Caspase-11 responds to non-classical activators rather than classic inflammasomes (Kayagaki et al., 2011). This macrophage death has been proven to be pyroptosis caused by the non-canonical inflammasome pathway, as the morphology of the dying macrophages resembles that of cells affected by cell death induced by caspase-1. Further research found that caspase-11 mediated pyroptosis by recognizing LPS in the cell wall of gram-negative bacterias (Aachoui et al., 2013b; Hagar et al., 2013; Kayagaki et al., 2013). Human’s caspase-4 and caspase-5 appear to have the same function as murine caspase-11, which is activated by binding to LPS directly. The response of caspase-11 and caspase-4/5 to LPS depends on the recognition of lipid A moiety by their caspase activation and recruitment domain (CARD) domain (Shi et al., 2014).

Activated caspase-4/5/11 cause pyroptosis by activating GSDMD. Unlike canonical signaling pathway, this caspase-4/5/11-mediated pyroptosis releases IL-1α and high mobility group box 1 (HMGB1) (Kayagaki et al., 2015; Cheng et al., 2017). Caspase-4/5/11 regulate the secretion of IL-1β by activating the NLRP3/ASC/caspase-1 pathway instead of maturing pro-IL-1β directly. Recent studies have shown that following intracellular LPS stimulation, caspase-11 is involved in the cleavage of pannexin-1 channels and ATP release, which finally activated the NLRP3/ASC/caspase-1 pathway (Kayagaki et al., 2015; Cheng et al., 2017).

### Cleaved Gasdermin Triggers Pyroptosis

GSDMD mainly contains two domains, the N-terminal gasdermin-N domain, which is the pore-forming fragment, and the C-terminal gasdermin-C domain, which inhibits the activation of gasdermin-N. These two domains are connected by a linker loop, and activated caspase-1 and caspase-4/5/11 cleave GSDMD at this linker region (Shi et al., 2015). The activation of gasdermin-N domain causes the perforation on membrane and eventually leads to pyroptosis. In intact GSDMD, the activity of gasdermin-N domain is inhibited due to its binding to gasdermin-C domain (Shi et al., 2015; Ding et al., 2016). The activated gasdermin-N domain can combine into oligomers and form pores in the cell membranes, including the plasma membrane. These membranes contain liposomes made of polar lipid mixtures, such as phosphoinositide and cardiolipin (Ding et al., 2016; Liu et al., 2016). Different from other pore-forming proteins, the asymmetric distribution of phosphoinositide on the plasma membrane leads to the specificity of GSDMD for mammalian cell pore formation (Aglietti et al., 2016; Sborgi et al., 2016).

GSDMD is a member of the gasdermin family, which is characterized by containing N domain. Gasdermin family proteins that have been discovered include GSDMA, GSDMB, GSDMC, GSDMD, GSDME (also known as DFNA5), and DFNB59 in humans (Shi et al., 2014). It is worth mentioning that mice do not possess GSDMB but have three subtypes of GSDMA and four subtypes of GSDMC. Except for DFNB59, the structures of GSDMA, GSDMB, GSDMC, and GSDME are similar to GSDMD, including the gasdermin-N domain and its binding inhibitory domain. Once activated, gasdermin-N domains can induce cell perforation and pyroptosis in mammalian cells (Kovacs and Miao, 2017; Feng et al., 2018). Due to the similar pore formation method of gasdermin-N domains, Shao group redefined pyroptosis as GSDM-mediated programmed cell death (Shi et al., 2017). This new definition suggests potential pyroptosis mechanisms in addition to the classical and non-classical pathways. Several recent studies found that Caspase-8 can also induce pyroptosis via directly cleaving GSDMD and GSDME (Orning et al., 2018; Sarhan et al., 2018).

## Pyroptosis in Atherosclerosis

Atherosclerosis is a chronic inflammatory disease involving multiple factors. Recruitment of monocytes/macrophages, proliferation and phenotypic transformation of SMCs and calcification constitute this pathological process (Bennett et al., 2016a). The balance of a variety of pro-inflammatory and anti-inflammatory factors, as well as ECs, macrophages and SMCs in plaques participate in the inflammation and death under various conditions, which are the focus of atherosclerosis research. Pyroptosis, a new cell death method, widely occurs in the initiation, progression and complications of atherosclerosis (Mensah, 2007; Zeng et al., 2019).

### Pyroptosis in the initiation of Atherosclerosis

**FIGURE 1:**
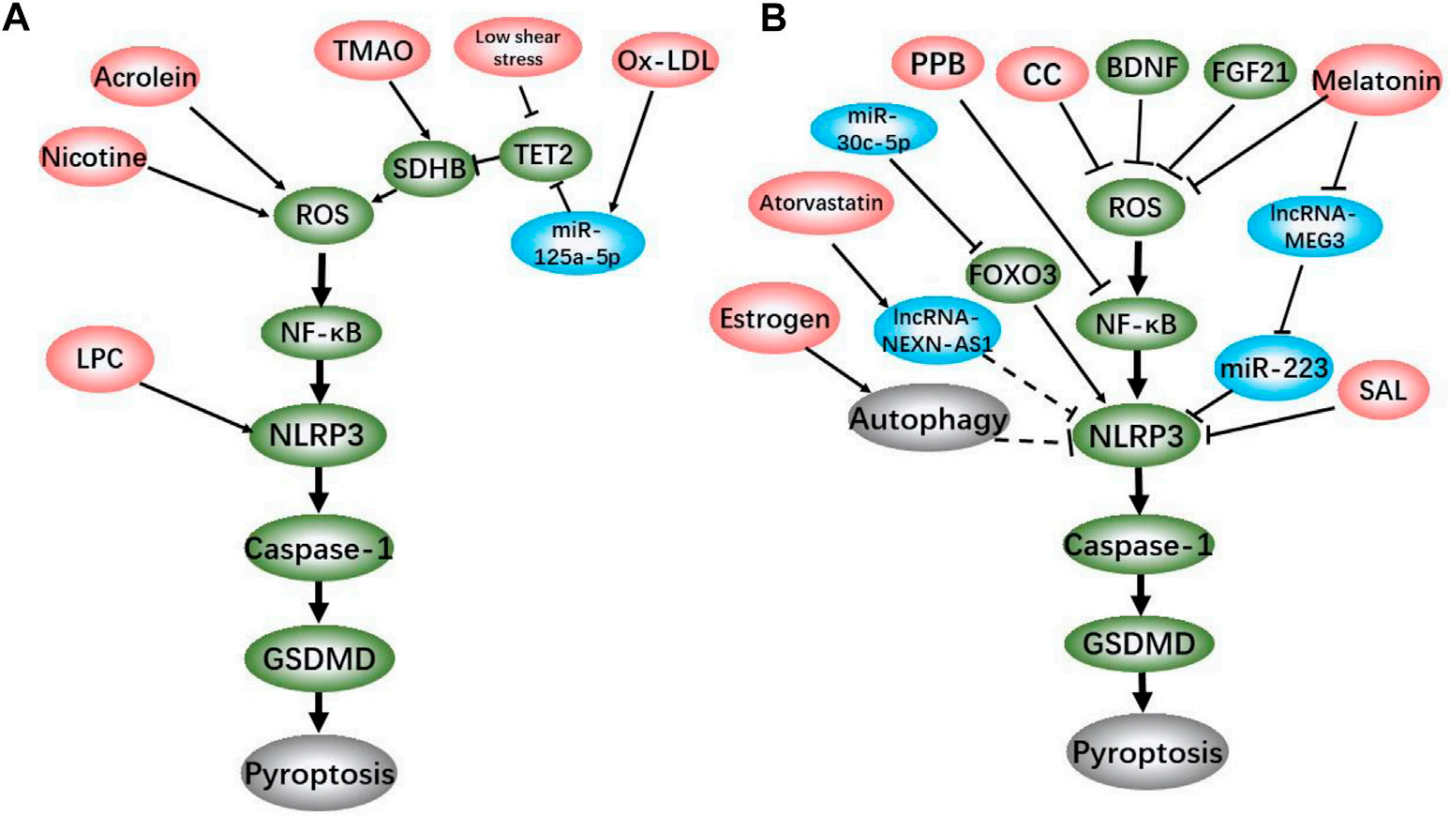
Positive factor **(A)** and Negative factor **(B)** regulating pyrotosis in ECs.

**FIGURE 2:**
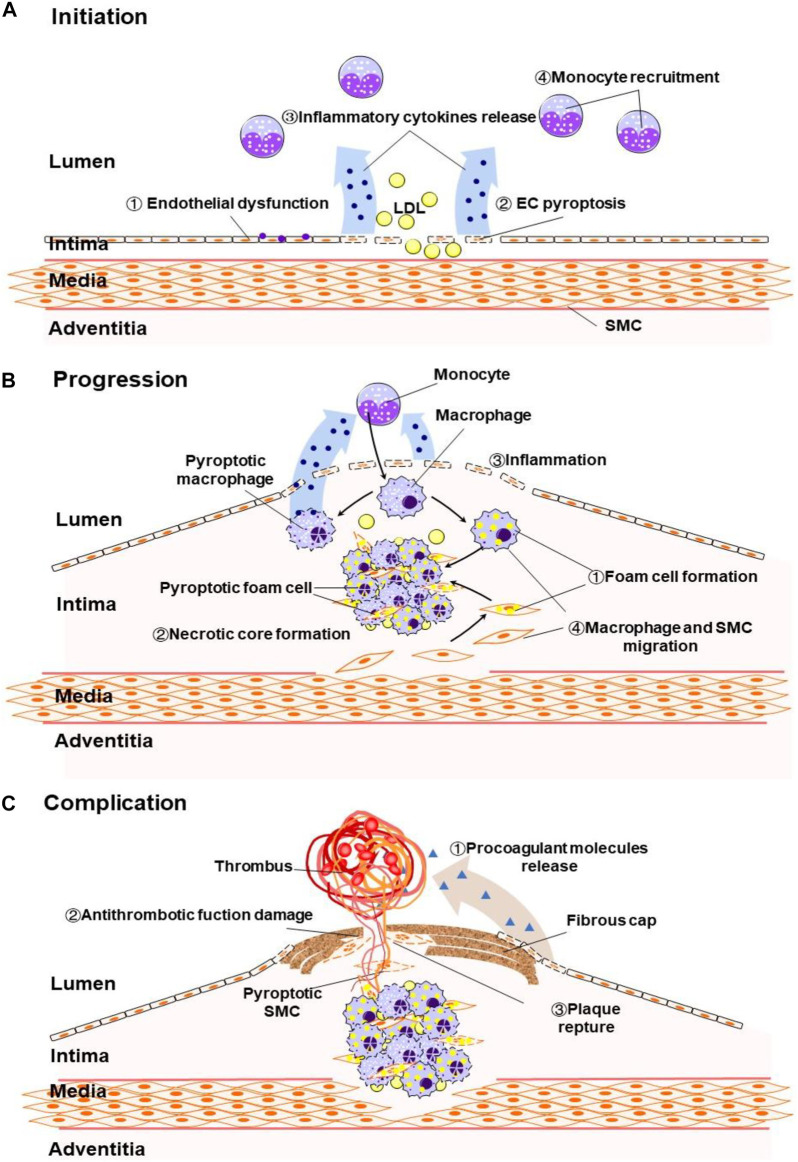
**(A)** Pyroptosis induce endothelial dysfunction, ECs death, inflammatory cytokines release and monocyte recruitment in at the early stage of atherosclerosis. **(B)** Pyrotosis induce foam cell formation and macrophage and SMC migration, increase inflammation and necrotic core formation in the advanced atherosclerosis. **(C)** Pyroptosis induce release of procoagulant molecules, thin the fibrous cap and promote plaque reptrue.

The dysfunction and death of ECs are the initiation of atherosclerosis (Libby et al., 2019). Under the action of serum inflammatory factors such as IL-1β, IL-18, tumor necrosis factor (TNF)-α and low density lipoprotein-cholesterol, ECs injury leads to the dysfunction and permeability change in vascular endothelial (Borén and Williams, 2016; Ference et al., 2017). Previous studies have classified death of ECs as cell necrosis or apoptosis, but with the reveal of new death mechanisms, it is detected that pyroptosis, a pro-inflammatory cell death, plays an important role in atherosclerosis. We summarized the atherosclerotic ECs pyroptosis induction/inhibitors and their brief mechanisms in the existing reports (Figure 1). The pyroptotic ECs cause endothelial dysfunction and release inflammatory cytokines to recruit monocytes (Figure 2A). Recent studies have shown that the NLRP3-mediated canonical pathway is the main mechanism of ECs pyroptosis in the initiation of atherosclerosis.

Yin and Wu reported that hyperlipidemia promotes ECs activation and death via caspase-1 activating in high-fat diet (HFD)-fed ApoE^−/−^ mice (Yin et al., 2015; Wu et al., 2018). Compared with ApoE^−/−^mice, the atherosclerotic area in the aortic sinus area of the caspase-1 and ApoE double knockout mice was reduced by 44% after 3 weeks after HFD feeding. The comparison of antibody arrays showed that the expressions of 17 cytokines and chemokines, such as intercellular adhesion molecule-1 (ICAM-1) and IL-1α, in ApoE^−/−^/caspase-1^−/−^ mice were lower than that in ApoE^−/−^ mice (Yin et al., 2015). Furthermore, it is reported that hyperhomocysteinemia and nicotine upregulate the expression of NLRP3, ASC, and Caspase-1 and induce death of ECs(Xi et al., 2016; Wu et al., 2018). Taken together, studies mentioned above suggest that these risk factors cause initiation of atherosclerosis via inducing ECs pyroptosis. Coincidentally, trimethylamine N-oxide (TMAO), an intestinal metabolite, treatment also confirmed the consistency between ECs pyroptosis and atherosclerosis in ApoE^−/−^ mice (Wu et al., 2020a).

On the other hand, existing studies have indicated that multiple drugs and genes such as melatonin, salidroside (SAL), estrogen, pyrogallol-phloroglucinol-6,6-bieckol (PPB) and fibroblast growth factor 21 (FGF21) decrease atherosclerosis plaque formation via inhibiting ECs pyroptosis *in vivo* and *in vitro* (Zhang et al., 2018; Oh et al., 2020; Xing et al., 2020; Zeng et al., 2020; Meng et al., 2021). Moreover, recent studis have discovered more drugs and miRNAs that could regulate ECs pyroptosis, including atorvastatin, miRNA-30c-5p, miRNA-125a-5p, etc. *in vitro* (Li et al., 2018; Zhaolin et al., 2019; Wu et al., 2020b). These laboratory work proved that the ECs pyroptosis is affected by complex factors. Although the role of these factors in atherosclerosis needs to be verified by further *in vivo* work, these discoveries have one thing in common that atherosclerosis-related ECs pyroptosis is achieved through the assembly of NLRP3 inflammasome and the activation of caspase-1.

With the continuous deepening of study, the mechanism of NLRP3-mediated ECs pyroptosis in the initiation of atherosclerosis has been gradually discovered. As a classic inflammatory signal, nuclear factor kappa-B (NF-κB) pathway has been proved to be involved in the pyroptosis of atherosclerotic ECs. Zeng et al. reported that after 2 h of NF-κB inhibitor BAY 11–7,082 treatment, the NLRP3, caspase-1 and IL-1β expression levels of ECs cultured with ox-LDL decreased significantly. PI staining results showed that BAY pretreatment also reduced the proportion of dead cells (Zhaolin et al., 2019). Correspondingly, studies showed that melatonin and PPB alleviate atherosclerosis in HFD-fed ApoE^−/−^ mice via inhibiting NF-κB-mediated pyroptosis in ECs(Schmitz and Ruebsaamen, 2010; Oh et al., 2020). It is well known that mitochondrial damage and reactive oxygen species (ROS) production are sufficient to activate NF-κB signal (Morgan and Liu, 2011; Formentini et al., 2012). By summarizing the existing data, we found the mechanisms of ox-LDL, nicotine, acrolein, TMAO, and blood low shear stress that promote NLRP3-mediated ECs pyroptosis are all related to mitochondrial damage and ROS production (Jiang et al., 2018; Wu et al., 2018). Succinate dehydrogenase complex subunit B (SDHB), located on the inner mitochondrial membrane, is a member of the succinate dehydrogenase (SDH) family, and plays a vital role in respiration and oxidation (Eng et al., 2003). The overexpression of SDHB promotes the ROS generation, while the transfection of SDHB shRNA abolishes the TMAO-induced pyroptosis in human umbilical vein endothelial cells (HUVECs) (Wu et al., 2020a). The down-regulation of SDHB inhibitory protein tet methylcytosine dioxygenase 2 (TET2) expression is involved in the pyroptosis caused by low shear stress and ox-LDL (Zhaolin et al., 2019; Chen et al., 2021). On the contrary, FGF21, brain-derived neurotrophic factor (BDNF) and colchicine (CC) effectively inhibit the ECs pyroptosis by stabilizing mitochondria and reducing ROS (Yang et al., 2020; Zeng et al., 2020; Jin et al., 2021). Various signs indicate that ROS/NF-κB/NLRP3 axis plays an important role in ECs pyroptosis in the initiation of atherosclerosis.

A recent study showed that estrogen inhibits ECs pyroptosis by inducing autophagy, thereby ameliorating atherosclerosis in mice. Estrogen reduced the expression of caspase-1 and GSDMD in homocysteine (Hcy)-treated HUVECs, while the autophagy inhibitor 3-MA reversed this effect (Meng et al., 2021). Meanwhile, Jiang et al. reported that autophagy alleviates the activation of NLRP3 inflammasome and pyroptosis caused by acrolein in ECs via improving mitochondrial function and reducing ROS (Jiang et al., 2018). Since ROS can activate NF-κB pathway (Evans and Salamonsen, 2012), it is speculated that the inhibition of pyroptosis by autophagy might be related to the ROS/NF-κB/NLRP3 axis, but this remains to be confirmed by further studies.

The latest research shows that nicotine activates bone marrow-derived macrophages (BMDMs) pyroptosis and BMDMs pre-treated with nicotine aggravate ECs apoptosis *in vitro*. Correspondingly, nicotine exacerbates mice endothelial damage and atherosclerosis, accompanied by enhanced macrophage chemotaxis *in vivo* (Mao et al., 2021). The results of this research also suggest that, atherosclerosis should be a complex pathological process that involves many kinds of cells. When studying the role of pyroptosis in the initiation of atherosclerosis, both the pyroptosis of ECs itself and other regulating cells should be considered.

### Pyroptosis in the Progression of Atherosclerosis

Macrophage-derived foam cells are the major cells involved in atherosclerosis lesions and a key factor in plaque instability (Moore et al., 2013). The death of macrophages modulates the development of atherosclerotic lesions. For example, in advanced lesions, macrophage death promotes necrotic core formation, increasing plaque vulnerability and thrombosis (Kockx and Herman, 2000; Seimon and Tabas, 2009). As a type of regulated necrosis that secretes pro-inflammatory factors, pyroptosis has been reported to account for a large part of the macrophages’ death in atherosclerotic plaques (Martinet et al., 2019). Pyrotosis in the plaque induces inflammation which causes macrophage and SMCs migration and promotes foam cell formation (Figure 2B). Integration of existing studies, it is proved that NLRP3-dependent macrophages and foam cell pyroptosis contributes to the progression of atherosclerosis. It is particularly noteworthy that Fidler et al. described the AIM2-dependent macrophage pyroptosis exacerbates atherosclerosis as well. According to their study, the Janus kinase 2 (Jak2) mutation Jak2^V617F^ in macrophages lead to DNA replication stress and activation of the AIM2 inflammasome, thereby aggravating formation of necrotic cores. Consistent with the knockout of AIM2, knockout of Caspase-1/11 or GSDMD alleviated atherosclerosis in mice Aim2 knockout in Jak2^V617F^mice (Fidler et al., 2021).

The formation of foam cells promotes the progression of atherosclerosis. Compared with high-concentration short-term stimulation, the low-concentration long-term ox-LDL treatment makes human monocyte-derived foam cells cultured *in vitro* prone to pyroptosis (Nogieć et al., 2020). As a major component of plasma membrane, lsophosphatidylcholine (LPC) is a critical factor with highly pro-inflammatory in ox-LDL’s atherogenic activity (Matsumoto et al., 2007). Corrêa et al. reported that LPC-induced foam cell formation in monocytes and ECs is dependent on caspase-1 activation and IL-1β release. Interestingly, it is showed that LPC induces the secretion of IL-1β in human monocytes, but not in ECs *in vitro* (Corrêa et al., 2019). Although further animal and clinical data are needed to support the above results, it is speculated that pyroptosis in monocytes/macrophages has a stronger pro-inflammatory effect than ECs. Futhermore, the pyroptosis in foam cells aggravates the progression of atherosclerosis by inducing inflammation.

A study of nicotine exacerbating atherosclerosis showed that increased intracellular ROS caused macrophages pyroptosis, which is manifested by the assembly of NLRP3 inflammasome, the cleavage of caspase-1 and the increase of IL-1β, IL-18, and GSDMD production (Mao et al., 2021). The nuclear translocation of p65, a key protein of the NF pathway, promotes the nicotine-induced pyroptosis in macrophages. When p65 is silenced with siRNA, the nicotine-induced protein expression of cleaved Caspase1, IL-1β, IL-18 are decreased remarkably in RAW264.7 cells (Xu et al., 2021). Moreover, experimental data confirms that ox-LDL activates the NLRP3-mediated pyroptosis pathway in macrophages (Lin et al., 2020; Mu et al., 2020). Peng et al. reported that mitochondrial outer membrane protein NIX inhibits ox-LDL-induced macrophages pyroptosis by activating mitophagy. Mitophagy inhibits the activation of caspase-1 and the maturation of IL-1β in macrophages by maintaining mitochondrial membrane potential and mitigated ROS production (Peng et al., 2020). It suggests that when stimulated by atherosclerotic risk factors, mitochondria have a mechanism to maintain its own stability to resist pyroptosis. Existing research results suggest that, similar to ECs, ROS/NF-κB/NLRP3 axis and mitochondrial damage are critical for macrophages pyroptosis in atherosclerosis.

Fortunately, with the studies of pyroptosis in atherosclerosis, progress has been made in the development of macrophages pyroptosis-related treatment methods. Han et al. reported that low dose sinapic acid inhibited the pyroptosis of bone marrow-derived macrophages in diabetic atherosclerotic rats and reduced the contents of serum IL-1β. It was also proved that low dose sinapic acid inhibits pyroptosis of high glucose-OxLDL treated macrophages via down-regulating the lncRNA-metastasis-associated lung adenocarcinoma transcript 1 (MALAT1) (Han et al., 2018). Piceatannol (PIC) is well-known as a cardioprotective drug. The latest research confirms that PIC up-regulates miR-200a and Nrf2 to inhibit macrophage pyroptosis and ultimately alleviate atherosclerosis (Mu et al., 2020). The study of bone marrow-derived mesenchymal stem cells microvesicles (BMSCs-MVs) on the treatment of atherosclerosis in mice showed that miR-223 inhibited macrophages pyroptosis by reducing the expression of NLRP3 (Lin et al., 2020). Non-coding RNA-dependent way will become the new treatment ideas of atherosclerosis.

### Pyroptosis in Complications of Atherosclerosis

Thrombosis triggered by tissue factors produced by macrophages and SMCs in plaques is the most serious complication of atherosclerosis (Libby et al., 2019). Under physiological conditions, the arterial endothelium possesses numerous properties that prevent clot formation and promote thrombolysis (Gimbrone and García-Cardeña, 2016). When endothelial dysfunction and inflammation occur, the endothelium loses its antithrombotic function and even releases the potent procoagulant molecules (Bevilacqua et al., 1986). Based on this feature, it can be inferred that the inflammatory factors released by the pyroptosis of cells in the plaque and the destruction of blood vessels can promote thrombosis, but this needs to be confirmed by more research data.

Atherosclerotic plaque rupture is a common cause of thrombosis that leads to myocardial infarction (Bentzon et al., 2014). SMCs migrate from the media layer to the intima layer and produce extracellular matrix to form a fibrous cap, both of which are indispensable in the progression of atherosclerosis (Bennett et al., 2016b). The thickness of the fibrous cap determines the stability of the atherosclerotic plaque, while inflammation attenuates the fibrous cap. The death of SMCs and the breakdown of extracellular matrix will make the fibrous cap thinner, which will eventually lead to plaque rupture, causing serious complications (Figure 2C) (Bennett et al., 2016a). It has been reported in the early years that the number of cells containing ox-LDL in plaques is positively correlated with the susceptibility to SMCs death (Akishima et al., 2005). As a proinflammatory form of death, pyroptosis may increase the instability of plaques and degrade the fibrous cap. A study showed that only a low concentration of ox-LDL stimulation is sufficient to transform SMC into a proinflammatory phenotype, which express more inflammatory factors and less contractile proteins (Kiyan et al., 2014). Ox-LDL can induce SMC pyroptosis by activating the NLRP3 inflammasome. As a specific inhibitor of caspase-1, VX-765 inhibits the pyroptosis of SMCs and slows down the progression of established plaques without influencing lipoprotein levels in plasma (Li et al., 2020). Moreover, Pan et al. reported that ox-LDL induces the caspase-1-mediated pyroptosis in SMCs through AIM2 inflammasome (Pan et al., 2018). This study suggests that in addition to NLRP3, there are other inflammasomes activating the atherosclerotic SMCs pyroptosis pathway. In general, existing studies have shown that ox-LDL increases the risk of atherosclerotic plaque rupture by activating SMCs pyroptosis. However, more evidence is needed to clarify the relationship between SMCs pyroptosis and the prognosis of atherosclerosis.

## Conclusion and Perspectives

As a proinflammatory form of regulated cell death, pyroptosis plays an important role in the initiation, progression and complications of atherosclerosis that involves ECs, pro-inflammatory leukocyte and SMCs. Pyroptosis of ECs and the initiation of atherosclerosis exacerbate vascular endothelial damage and dysfunction by inducing the release of proinflammatory cytokines, such as IL-1β and IL-18. These cytokines can promote the migration of bound T lymphocytes and monocytes to early plaques (Libby et al., 2019). Pyroptosis of macrophages further releases cytokines and promotes the formation of foam cells. The SMCs in the medium can migrate to the inner membrane in response to the mediators produced by endothelial dysfunction and LDL accumulation. Pyroptosis of SMCs decreases the size of the fibrous cap by causing the loss of collagen and matrix, promoting plaque instability, and even plaque rupture (Figure 2).

Currently, there are still some limitations in the studies of pyroptosis in atherosclerosis. Firstly, most of the experimental work focuses on the ECs pyroptosis in the initiation of atherosclerosis. In the last two years, there have been more reports that the pyroptosis of macrophages and foam cells exacerbates the progression of atherosclerosis. But pyroptosis in SMC, which is an important factor affecting plaque stability, is relatively less reported. This may due to the difficulty of either establishing experimental models or identifying the source of cells in plaques. Fortunately, with the development of single-cell sequencing technology, various cells in plaques can be accurately located and identified, making it possible to establish experimental models for the distinguishing of the source of participant cells (Allahverdian et al., 2018; Wirka et al., 2019). Secondly, many studies still require further *in vivo* data to confirm the role of pyroptosis in atherosclerosis. Atherosclerosis is a complex pathological process involving multiple cells, so the results of a single type of cell *in vitro* can hardly explain the actual disease *in vivo*. Although some studies have used the model of ApoE mice, there are no reports that include clinical data. Compared with hard-to-obtain plaque tissue samples, detecting the level of pyrolysis-related molecules and the pyroptosis of leukocytes in the peripheral blood of patients it is a good starting point. Thirdly, the selection of inducers for the positive control group of pyroptosis in some studies is open to discussion. The combination of LPS and ATP is a common pyroptosis inducer, but LPS does not exist in the process of atherosclerosis without co-infection. Atherosclerotic risk factors such as ox-LDL are a more suitable for positive control. Furthermore, existing studies focus on the function of single protein or non-coding RNA, but rarely involve the complete signal pathway. This makes our understanding of pyroptosis in atherosclerosis tend to be fragmented. The ROS/NF-κB/NLRP3 axis speculated in this review is a feasible idea, and we hope that more hypotheses will be proposed.

In this review, we summarized the stimulators and drugs that promote/inhibit pyroptosis in atherosclerosis of related reports (Table 1). Ox-LDL, acrolein, TAMO, LPC, low shear stress, etc. are risk factors for atherosclerosis, among which Ox-LDL is the most critical. Due to the biological characteristics of atherosclerotic risk factors, the pyroptosis of cells in lesions mainly depends on the activation of NLRP3 inflammasome that detects DAMPs. Most of these studies on NLRP3-mediated pyroptosis in atherosclerosis focus on the detecting of pyroptosis, while ignoring the underlying mechanism which causes the activation of inflammasomes. Based on the analysis and summary of the existing reports, it is found that mitochondrial damage, intracellular ROS production and activation of NF-κB pathway are the potential mechanisms of pyroptosis in atherosclerosis. We speculate that the ROS/NF-κB/NLRP3 axis may play an important role in atherosclerosis-related pyroptosis, meanwhile TET2/SDHB may be a potential regulatory factor, further research will be of great significance. AIM2-related SMCs and macrophages pyroptosis promoting plaque formation in mice further indicates the complexity of the pyroptosis mechanism in the process of atherosclerosis. Future studies should focus on the regulatory mechanism of NLRP3-mediated pyroptosis and the possible role of other inflammasomes in atherosclerosis. In related mouse experiments, GSDMD knockout reduce necrotic core formation, suggesting the application value of GSDMD knockout animal model and the potential treatment of atherosclerosis by GSDMD inhibitors.

**TABLE 1:** Mechanisms of drugs and stimulators regulating pyroptosis in the atherosclerosis. White: Positive factors of pyrotosis. Gray: Negative factors of pyrotosis.

Stimulators/drugs	Cell types	Targets/mechanisms	Proof *in vivo*	References
Melatonin	ECs	lncRNA-MEG3/miR-223	ApoE^−/−^ mice	Zhang et al. (2018), Wang et al. (2019)
		ROS/NLRP3	SD rats	
Atorvastatin	ECs	lncRNA-NEXN-AS1/NLRP3	/	Wu et al. (2020b)
SAL	ECs	NF-κB/NLRP3	ApoE^−/−^ mice	Xing et al. (2020)
FGF21	ECs	ROS/NLRP3	ApoE^−/−^ mice	Zeng et al. (2020)
BDNF	ECs	KLF2/HK1, ROS/NLRP3	/	Jin et al. (2021)
Colchicine	ECs	AMPK/STRT/ROS	/	Yang et al. (2020)
Estrogen	ECs	Autophagy	ApoE^−/−^ mice	Meng et al. (2021)
PPB	ECs, SMCs	TLR4/NF-κB/NLRP3	/	Oh et al. (2020)
SA	Macrophages	lnc-MALAT1/NLRP3	Diabetic rats	Han et al. (2018)
NIX	Macrophages	Autophagy	/	Peng et al. (2020)
Piceatannol	Macrophages	miR-200a/Nrf2	/	Mu et al. (2020)
BMSCs-MVs	Macrophages	miR-223/NLRP3	/	Lin et al. (2020)
HDAC6	Macrophages	NF-κB/NLRP3	ApoE^−/−^ mice	Xu et al. (2021)
Acrolein	ECs	ROS/NLRP3	/	Jiang et al. (2018)
TMAO	ECs	SDHB/ROS	ApoE^−/−^ mice	Wu et al. (2020a)
Low shear stress	ECs	TET2/SDHB/ROS	/	Chen et al. (2021)
LPC	ECs, monocytes	NLRP3	/	Corrêa et al. (2019)
Jak2 mutation	Macrophages	Jak2/AIM2/GSDMD	Casp1/11^−/−^, GSDMD^−/−^, Jak2^VF^Casp1/11^−/−^, Jak2^VF^Aim2^−/−^ and Jak2^VF^Nlrp3^−/−^ mice	Fidler et al. (2021)
Ox-LDL	ECs	miR-125a-5p/TET2/SDHB	/	Zhaolin et al. (2019) Nogieć et al. (2020)
	ECs,macrophages	ROS/NF-κB	/	
	SMCs	TLR4/NF-κB	/	Kiyan et al. (2014)
		AIM2/GSDMD	ApoE^−/−^ mice	Pan et al. (2018)
Nicotine	ECs macrophages	ROS/NF-κB	ApoE^−/−^ mice	Wu et al. (2018)
		TXNIP/NLRP3	ApoE^−/−^ mice	Mao et al. (2021)

Research on pyroptosis-related drugs shall provide new ideas for the treatment of atherosclerosis in the future. Traditional cardiovascular drugs such as PIC have been proven to alleviate atherosclerosis by suppressing pyroptosis. The role of some non-coding RNAs such as miR-223, miR-30–5p, and lncR-MALAT1 in pyroptosis has been revealed to provide new targets for the treatment of atherosclerosis. More *in vivo* studies and clinical trials are required to provide a basis for the development of pyroptosis targeted drugs.
